# Microwave Absorption Properties of Magnetite Particles Extracted from Nickel Slag

**DOI:** 10.3390/ma13092162

**Published:** 2020-05-07

**Authors:** Pengze Yan, Yongqian Shen, Xueyan Du, Junkai Chong

**Affiliations:** State Key Laboratory of Advanced Processing and Recycling of Nonferrous Metals, School of Materials Science and Engineering, Lanzhou University of Technology, Lanzhou 730050, Gansu, China; yanpz@lut.edu.cn (P.Y.); syqch@lut.edu.cn (Y.S.); chongjk@lut.edu.cn (J.C.)

**Keywords:** nickel slag, magnetite, ball-milling, interfacial polarization, microwave absorption

## Abstract

The utilization of nickel slag has attracted much attention due to its high-content of valuable elements. As a part of these efforts, this work focuses on whether magnetite crystals, obtained from nickel slag via molten oxidation, magnetic separation, and ball-milling can be used as a microwave absorber. The composition, morphology, microstructure, magnetic properties, and microwave absorption performance were characterized by X-ray diffraction (XRD), X-ray photoelectron spectroscopy (XPS), field emission scanning electron microscopy (FE-SEM), transmission electron microscopy (TEM), vibrating sample magnetometer (VSM), and vector network analysis (VNA). The results reveal that the magnetite crystals exhibit excellent microwave absorption properties because of the synergistic action between dielectric loss and magnetic loss. The minimum reflection loss (RL) of the particles obtained after 6 h ball-milling reaches −34.0 dB at 16.72 GHz with thickness of 5 mm. The effective frequency bandwidth (RL ≤ −10 dB) is 4.8–5.4 GHz and 15.9–17.6 GHz. Interfacial polarization of the particles could play a crucial role in improving absorbing properties because several components contained in the particles can dissipate electromagnetic wave effectively. The current study could show great potential in the preparation of magnetite crystals and utilization of nickel slag.

## 1. Introduction

As the demand for information technology and electronic equipment continues to rise, the utilization of high-performance broad-band microwave absorbing materials (MAM) becomes more critical in decreasing or even eliminating electromagnetic (EM) pollution, which impacts human health and disrupts electronic equipment functioning [1,2,3,4]. Microwave energy dissipates when an EM wave interacts with an MAM, which then converts this energy into another one (e.g., thermal) through its own electric and magnetic losses. However, achieving best performance for material containing only a single component is difficult because of impedance mismatch [5,6].

Magnetite is very attractive as an MAM because of its low cost and excellent magnetic properties. However, its direct use is difficult because of an impedance mismatch and poor antioxidation. Therefore, to solve this problem, magnetite can be combined with dielectric materials, such as SiO_2_, ZnO, SnO_2_, and MnO_2_ [7,8,9,10,11]. The results indicate that the synergistic action between magnetite and dielectric substance enhances microwave absorption performance, reducing the absorbing thickness of the materials. However, this approach has certain disadvantages, such as rigorous reaction conditions and the need to retrieve organic wastewaters generated after their synthesis, which restricts its broader application in the industry [12].

Nickel slag is produced during the nickel metallurgy process as industrial solid waste. The large amount of nickel slag in the environment is a serious problem, resulting from largescale occupation of farmland and causing secondary environmental pollution [13,14]. Thus, nickel slag utilization and recycling have recently attracted attention, with special focus on the extraction of valuable metallic elements and their reuse. For instance, the recovery of iron resources from nickel slag using oxidation and magnetic separation with optimal control of reaction conditions has been reported [15]. Shoya et al. fabricated self-compacting concrete using nickel slag, exhibiting excellent performance and durability [16]. However, these techniques are somewhat costly and difficult to use in industrial applications on a large scale [17]. Therefore, it is necessary to find a practical and effective method to realize the high-valued utilization of nickel slag.

Magnetite particles (MPs) obtained from the oxidized nickel slag possess strong magnetic properties and hetero-interfaces formed by impurities, which are expected to be helpful in improving EM wave absorption. So far, however, there has been little discussion on the EM wave absorption of MPs gained from molten slag. Furthermore, considering microwave absorption strongly correlates with particle size [18], we conduct ball milling to enhance the EM wave properties of MPs since ball milling can conveniently control the size of MPs [19]. At the same time, the silicates can strip off during the ball milling process, resulting in the enhancement of the MPs’ magnetic properties. Accordingly, this work reports a low-cost and environment-friendly design to prepare MAM. The microwave absorption properties of magnetic particles milled at different conditions were tested in the range of 2.0–18.0 GHz, and the mechanism of EM wave loss was elaborated upon.

## 2. Experimental

### 2.1. Materials

The raw material was water-quenched nickel slag, acquired from flash furnaces in Jinchuan Group (Jingchang, China). Its composition is given in Table 1. Ethanol was purchased and used without any further purification. The crucibles used in this work were fabricated using 99% pure alumina.

### 2.2. Experimental Procedures

Iron recovered from the Ni-slag was oxidized into magnetite and then magnetically separated to obtain MIPs. For this purpose, Ni-slag was crushed into particles with size below 74 μm, then placed into an alumina crucible, and heated to 1450 ℃ for 30 min. The oxidized slag was then subjected to magnetic separation. 200 mesh MPs (marked as sample M0) were then ball milled at 300 rpm for 3 h (sample M1), 6 h (sample M2), and 12 h (sample M3), respectively. The ball-to-powder ratio was 30:1. After the ball milling, the resulting material was washed several times with ethanol and then dried at 60 °C for 12 h in a vacuum furnace.

### 2.3. Characterization

X-ray diffraction (XRD) patterns were recorded on the D8 ADVANCE instrument (Bruker AXS, Karlsruhe, Germany) with Cu-Kα radiation (λ = 1.5418Å) as an X-ray source. Crystal morphology and elemental analysis were obtained using an electron probe microanalysis (EPMA) performed using the EPMA-1600 instrument (Shimadzu, Kyoto, Japan). X-ray photoelectron spectroscopy (XPS) measurements were performed on PHI-5000 Versa probe instrument (Ulvac-Phi, Kanagawa, Japan). Field emission scanning electron microscopy (FE-SEM), transmission electron microscopy (TEM), high resolution transmission electron microscopy (HRTEM), and selected area electron diffraction (SAED) images were collected on JSM-6700F (JEOL, Tokyo, Japan) and JEM-2100 (JEOL) instruments, respectively. Room temperature magnetic hysteresis loops were conducted by vibrating sample magnetometer (VSM), Lake Shore 7307 (Lake Shore, Westville, NJ, USA). Electric resistivity was measured by a four-probe method using a double electrical RTS-9 tester (Nuoleixinda Co., Ltd., Tianjin, China). EM parameters were tested by a vector network analysis (VNA) coupled with a coaxial line method performed on the Agilent N5244A instrument (Agilent, California, CA, USA). All the powders were uniformly dispersed in paraffin matrix with a filling ratio of 70 wt% and pressed into a toroidal ring with outer diameter of 7.00 mm, inner diameter of 3.04 mm, and thickness of 2.00 mm. Complex permeability and permittivity were obtained from experimental scattering parameters according to the standard Nicolson-Ross and Weir theoretical calculations.

## 3. Results and Discussion

**Scheme 1:**
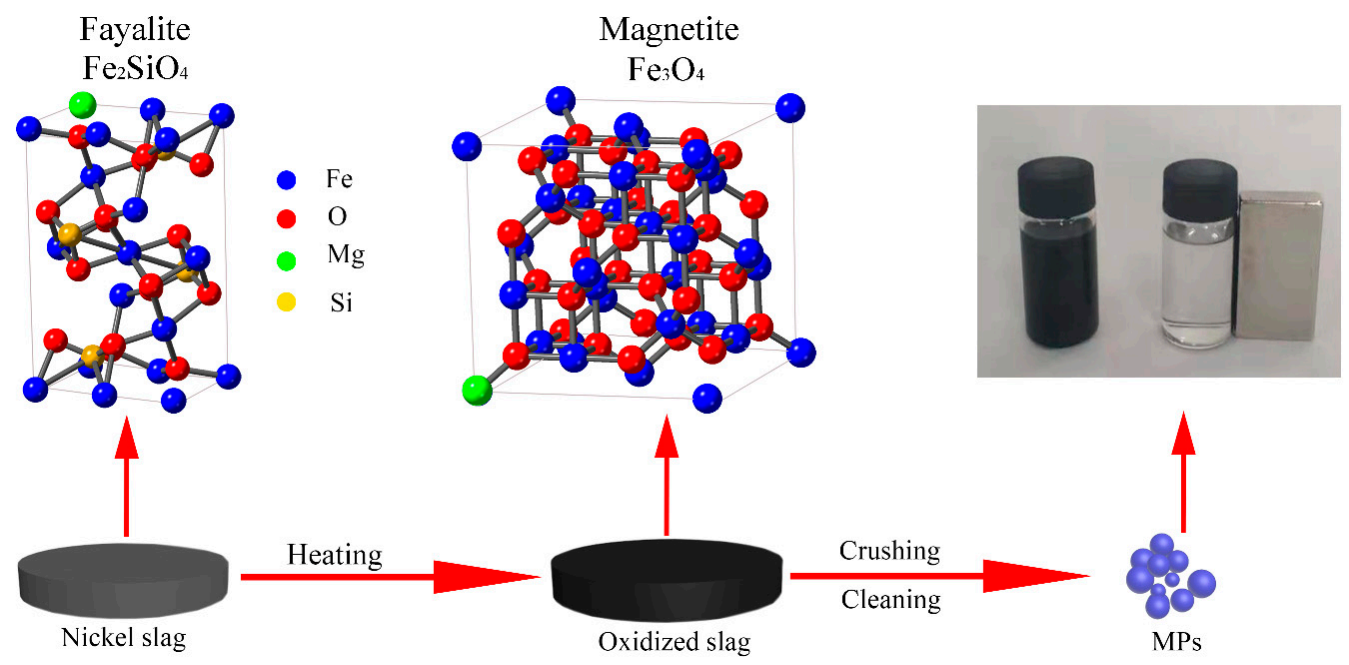
Schematic illustration of the formation of magnetite particles (MPs).

Nickel slag was crushed into particles with specific size and then oxidized during the heating process, causing magnetite formation, as shown in Scheme 1. During this process, fayalite was transformed into magnetite. Mg^2+^ from the nickel slag was able to dissolve and replace Fe^2+^ in magnetite.

### 3.1. Structure Characterization

**Figure 1:**
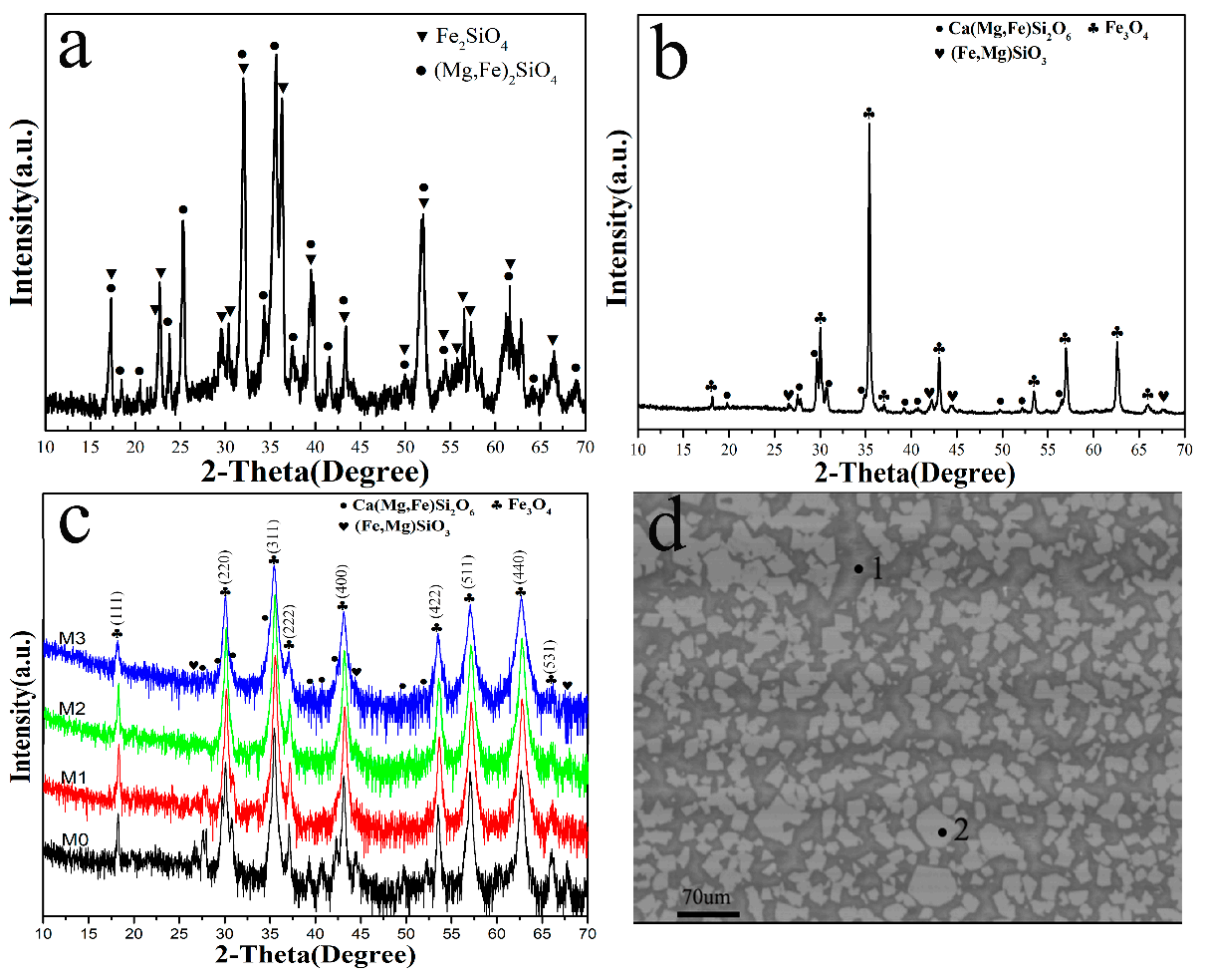
XRD pattern of nickel slag (**a**), XRD pattern of oxidized nickel slag (**b**), XRD patterns of MPs with different milling time (**c**), and backscattering electron image of oxidized nickel slag (**d**).

Fayalite, the most common iron-bearing phase in Ni-slags, transforms to magnetite below 1723 K, as shown in Figure 1a,b. Particle size directly affects the EM wave absorption properties of a material [20]. Thus, the obtained MPs were ball milled to analyze their EM wave absorption properties. XRD patterns of the ball-milled samples are shown in Figure 1c, where the primary peaks at 18.2°, 30.1°, 35.4°, 37.1°, 43.1°, 53.5°, 57.0°, and 62.6° can be assigned to (110), (220), (311), (222), (400), (422), (511), and (440) lattice planes, respectively, which are well consistent with the cubic inverse spinel structure of magnetite (JCPDS card No. 19-0629). The XRD pattern of the M0 sample shows a silicate peak appearance, which, however, is not observed in the diffraction patterns of the M1, M2, and M3. This is probably attributable to the peeling of silicates from magnetite after ball-milling and magnetic separation treatment. With mill time increasing, the diffraction peaks associated with magnetite become broadened due to the decrease in grain size and increase in internal stress [21]. Besides, the crystallinity of the diffraction peak at 35.4° for all samples have been calculated. The crystallinity of the M0 was found to be about 35.4%. It decreases to 34.1% and 32.4% after the 3 h and 6 h ball-milling, respectively. When the ball milling time prolongs to 12 h, the crystallinity drops down to 27.1%.

The Hall-Williamson equation [22,23] is often used to calculate the average internal strain and grain size of the powders:

(1) $$\beta hkl={\left[{\left(\beta hkl\right)}^{2}measured-{\left(\beta hkl\right)}^{2}instrumental\right]}^{\frac{1}{2}}$$

(2) $$\beta_{hkl}\cos\theta hkl=\frac{k\lambda }{\delta }+4\varepsilon sin\theta hkl$$

where *β_hkl_* is the full width at the half-maximum (FWHM) of the corresponding diffraction peaks, (*β_hkl_*_, *measured*_ is the measured value, while *β_hkl, instrumental_* is the eigenvalue of the instrument), *θ* represents the Bragg’s angle, *k* is the shape factor (equal to 1), *λ* stands for the X-ray wavelength, *δ* means the grain size, and *ε* is the internal stain.

The grain size decreases slightly during the early stages of the ball-milling process due to the silicate separation from the samples, as shown in Table 2. It can be observed that grain size reduces with increase in ball-milling time and quickly decreases in the range of 6 h to 12 h, while the internal strain increases gradually with prolonging of ball-milling time.

The backscattering electron image of the oxidized nickel slag (shown in Figure 1d) demonstrates that the main phases in areas marked with 1 and 2 are assigned to silicate and magnetite, respectively, according to data analysis in Table 3. Mg and Si content in point 2 were 3.1 and 0 wt%; implying that Mg incorporates into magnetite lattice during high-temperature treatment. This seems reasonable since the ionic radius of Fe^2+^ approaches that of Mg^2+^.

**Figure 2:**
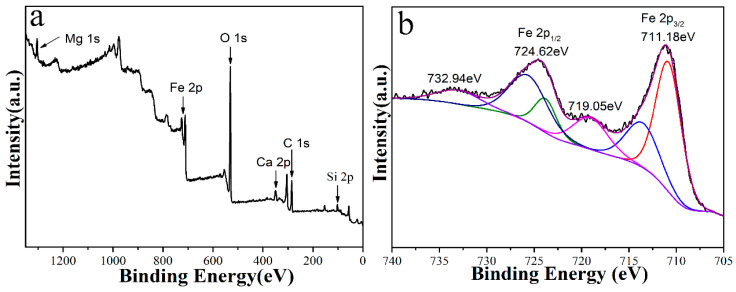
XPS spectra of M2: wide scan (**a**); high-resolution Fe 2p (**b**).

XPS analysis shows the presence of Mg, Fe, O, Ca, and Si in the M2 sample (see Figure 2a), which indicates that silicates are only partially separated from magnetite during the ball-milling process. The Fe 2p spectrum can be divided into six peaks (see Figure 2b). The peaks at 711.07 and 723.69 eV attribute to Fe^2+^ in Fe_3_O_4,_ while peaks at 714.18 and 725.57 eV are assigned to Fe^3+^ in Fe_3_O_4_. The satellite peak at 719.05 eV indicates the co-existence of Fe^2+^ and Fe^3+^ [24]. Another satellite peak is probably attributed to other unidentified Fe compounds [25].

### 3.2. Morphology Analysis

**Figure 3:**
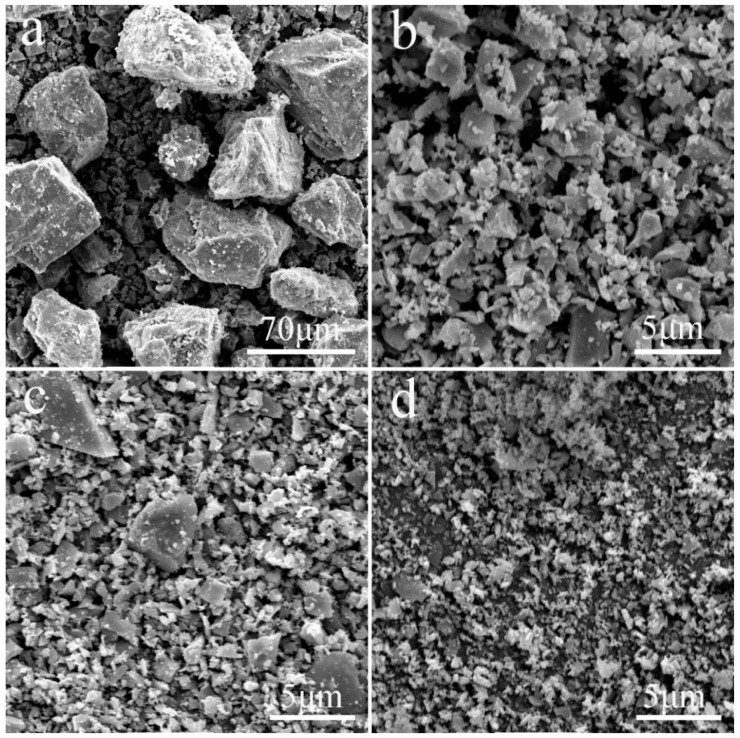
SEM images of MPs with different ball-milling time: 0 h (**a**), 3 h (**b**), 6 h (**c**), and 12 h (**d**).

**Figure 4:**
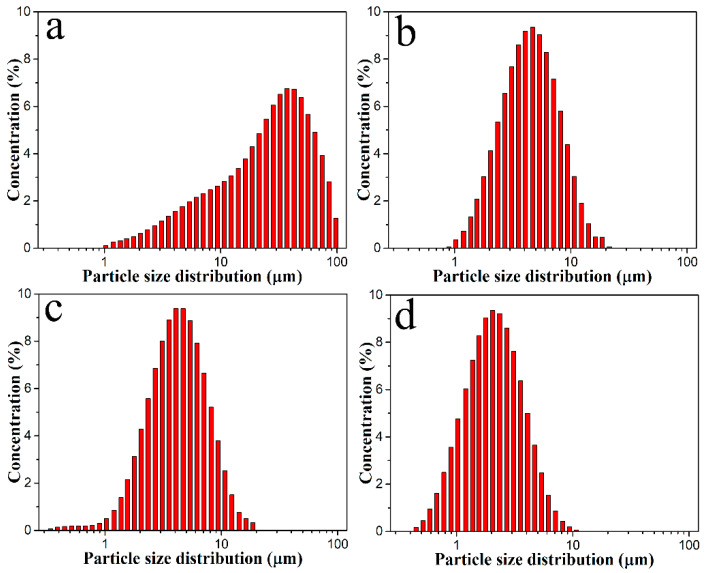
Particle size distribution for M0 (**a**), M1 (**b**), M2 (**c**), and M3 (**d**).

**Figure 5:**
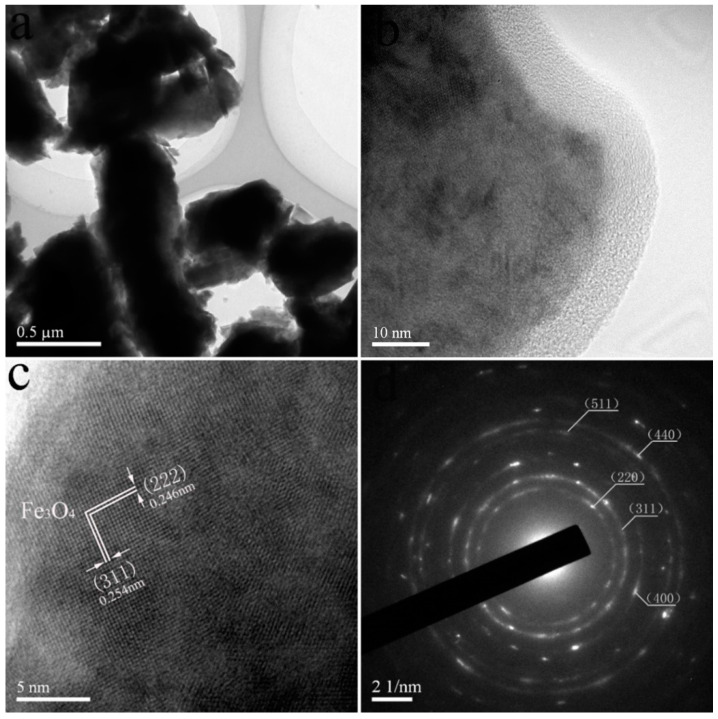
Different magnification TEM images (**a**,**b**), HRTEM image (**c**), and SAED pattern (**d**) of M3.

The particle size of the M0 sample is in the range of 20–60 μm (see Figure 3 and Figure 4). It can be seen that most of these particles exhibit irregular shapes and the ball milling effectively reduces particle size. Even though ball milling is beneficial in improving some properties, overextended milling has an alternative effect, for example, particles milled for 12 h start to agglomerate (see Figure 3d) owing to the increase in particle surface energy. Particle size distribution with different ball milling times indicates that the ball milling method can effectively tailor particle size and improve its distribution, as shown in Figure 4. TEM images of sample M3 show that particles possess irregular shapes and slight agglomeration, in the size range of 0.1–1 μm, as shown in Figure 5a. Besides, there is an amorphous layer on the surface of magnetite particles, as shown in Figure 5b, which could be a result of excessive ball milling. HR-TEM results demonstrate the lattice fringes to be 0.246 and 0.254 nm apart, which correspond to the (222) and (311) planes of magnetite, respectively (see Figure 5c). SAED data shows the diffraction rings assigned to the (220), (311), (400), (511), and (440) planes of magnetite (see Figure 5d), which agrees well with the XRD results:

### 3.3. Magnetic Analysis and Microwave Absorption Properties

**Figure 6:**
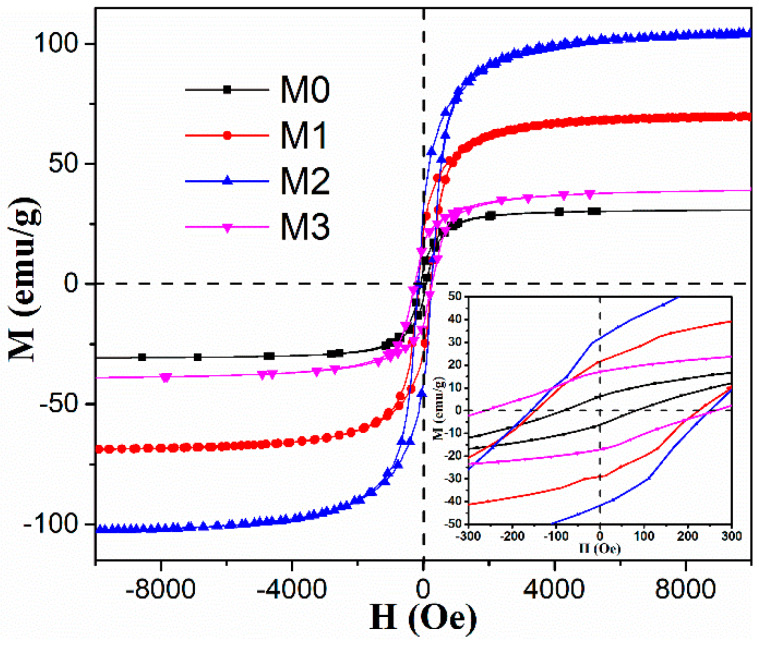
Hysteresis loops of the M0, M1, M2, and M3. The insert is the magnified loop.

The magnetic properties of materials directly correlate with their ability to absorb electromagnetic wave energy. Figure 6 shows the hysteresis loops of the as-prepared samples. The saturation magnetization (*Ms*) is about 30.8 emu/g for M0, and increases to 70.4 emu/g for M1 and up to 103.4 emu/g for M2. This is probably attributed to the formation of small clusters with high *Ms*, for example, α-Fe_3_Si has a *Ms* of 150 emu/g. On the contrary, *Ms* of the M3 decreases down to 39 emu/g, which is mainly ascribed to its increasing defects density and the amorphous layer on its surface [26,27].

The coercivity forces (H_c_) of M0, M1, M2, and M3 samples are 83.4, 187.3, 203.3, and 264.2 Oe, respectively. The M3 sample shows the highest H_c_ value, resulting from the increase of defect density and decrease in grain sizes, which cause the material to become hard to magnetization for the pinning effect [28,29].

**Figure 7:**
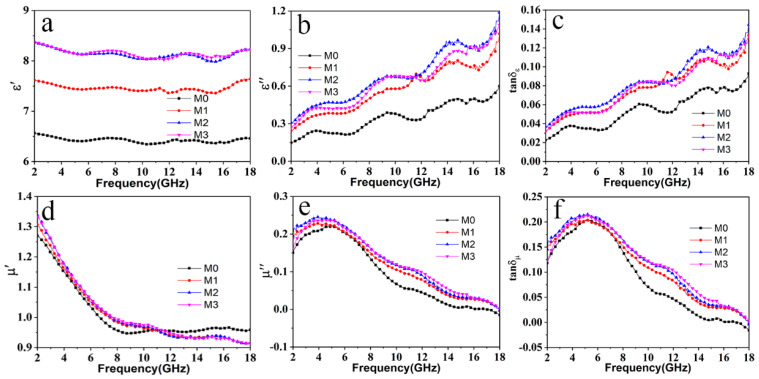
The real part of complex permittivity (**a**), the imaginary part of complex permittivity (**b**), dielectric loss tangent (**c**), the real part of complex permeability (**d**), the imaginary part of complex permeability (**e**), magnetic loss tangent (**f**).

Materials’ EM wave absorption properties depend on their complex permittivity and permeability. The real and imaginary parts of the complex permittivity and permeability represent the storage and loss capabilities of the electric and magnetic energies, respectively. The EM parameters of the composites containing 70 wt% paraffin show that both *ε′* and *ε″* values rise with prolonging of ball-milling time (see Figure 7a,b). This mainly results from smaller particle sizes and electrical conductivity increase [21,30]. According to the free-electron theory [31], *ε″* can be expressed as:

(3) $$\varepsilon {}^{\prime \prime }\approx 1/2\varepsilon_{0}\pi ƒ\rho$$

where *ρ* is the resistivity, *ε*_0_ is the permittivity in a vacuum, and *ƒ* is microwave frequency. Larger *ε″* values indicate lower electrical resistivity. To verify this expression, the electrical resistivity of M1, M2, and M3 was performed; the results are shown in Table 4. M2 shows the lowest resistance values and the highest *ε″* values. Moreover, multiple dispersion peaks appear in Figure 7b, which is probably owing to the heterogeneous interface in this sample [32].

Figure 7d,e give the *μ′* and *μ″* values in the frequency range of 2.0–18.0 GHz. With the frequency increasing, the *μ′* values first decrease for all samples due to ferromagnetic resonance and eddy current loss, and then stay nearly unchanged. The *μ″* values increase first and then decrease. Generally, *µ_i_* is closely related to the complex permeability of ferromagnetic materials, *µ_i_* can typically be expressed as follows [33]:

(4) $$\mu_{i}=\frac{M_{s}{}^{2}}{akH_{c}M_{s}+b\lambda \xi }$$

where *μ_i_* is the initial permeability, a and b are the constants related to the material, *λ* is the magnetization constant, and *ξ* is the elastic strain parameter. The *μ_i_* value represents the magnetic loss ability of EM wave. Higher *M_S_* and lower H_c_ values are thus needed to improve *μ_i_* according to Equation (4). Therefore, M2 has the highest *μ*″ value in the 2.0–8.0 GHz. The variation of dielectric and magnetic loss tangents (tan *δ_ε_* = *ε″/ε′* and tan *δ_μ_* = *μ”*/*μ′*, respectively) are shown in Figure 7c, f, respectively. The tan *δ_μ_* value is larger than the tan *δ_ε_* value at low frequencies, indicating that magnetic loss is stronger than dielectric loss for all samples. However, dielectric loss prevails in the 12.0–18.0 GHz.

**Figure 8:**
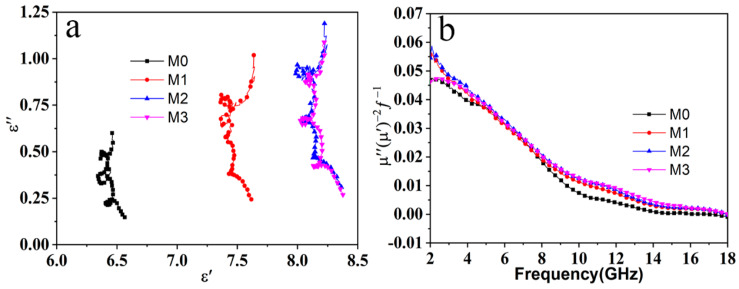
Cole−Cole semicircles (**a**), the curves *of μ″(μ′)^−^*^2^*f^−^*^1^ versus frequency (**b**) for M0, M1, M2, and M3.

Many studies have reported that Debye dipolar relaxation can promote EM wave absorption. The relationship between ε′ and ε″ can be described by the Debye theory [34] as follows:

(5) $$\left(\varepsilon {}^{\prime }-\frac{\varepsilon_{s}+\varepsilon_{\infty }}{2}\right)+{\left(\varepsilon {}^{\prime \prime }\right)}^{2}={\left(\frac{\varepsilon_{s}-\varepsilon_{\infty }}{2}\right)}^{2}$$

where *ε_s_* and *ε_∞_* are the static dielectric constant and the dielectric constant at an infinite frequency, respectively. Thus, *ε′* plotted as a function of *ε″* would be a single semicircle, known as a Cole-Cole semicircle. Each semicircle represents a Debye dipolar relaxation. Experimentally obtained *ε′* values plotted as a function of the corresponding *ε″* values for M0, M1, M2, and M3 are given in Figure 8a, indicating the existence of the dielectric relaxation in these samples [35]. As the ball-milling time prolongs, the curve shifts to the right. At the same time, the semicircle diameters increase, which indicates an increasing effect of quadratic dielectric relaxation on the dielectric constant. Thus, the ball milling process can control the Debye relaxation by the adjustment of particle size.

Magnetic loss also plays a crucial role in EM wave absorption. The natural and exchange resonances, as well as eddy current loss, contribute to magnetic loss in the GHz region. The eddy current losses can be expressed by the following equation [36]:

(6) $$\mu {}^{\prime \prime }{\left(\mu {}^{\prime }\right)}^{-2}{ƒ}^{-1}=2\pi \mu_{0}\sigma d^{2}/3$$

where *μ*_0_ is the permeability in a vacuum, and *ƒ* is frequency. *(μ″(μ′)^−^*^2^*ƒ^−^*^1^*)* in Equation (6) would be constant if the magnetic losses are mostly caused by Eddy current losses. This phenomenon can be found after 16 GHz in Figure 8b, which means that Eddy current loss exists in the as-prepared samples. The presence of two resonance peaks at 6.1 and 12.8 GHz, according to Aharoni’s theory [37], are associated with natural and exchange resonance, respectively.

**Figure 9:**
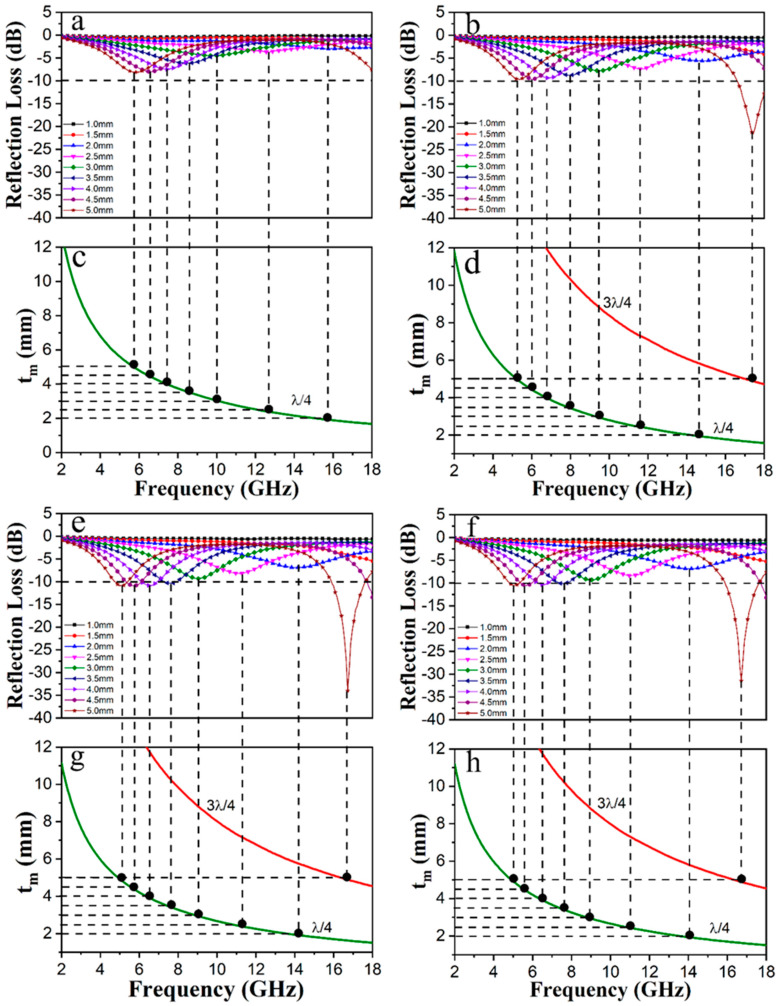
Reflection loss curves of M0 (**a**), M1 (**b**), M2 (**e**), and M3 (**f**). Dependences of matching thickness (*t_m_*) on matching frequency (*ƒ_m_*) of M0 (**c**), M1 (**d**), M2 (**g**), and M3 (**h**) simulated with the λ/4 model.

To further study microwave absorption performance, we calculated reflection loss (RL) values for our samples using the transmission line model. Based on the model of the metal-backed materials acting as a microwave-absorption layer, the reflection loss (RL) values were calculated according to the transmission-line theory by Equations (7) and (8) [38]:

(7) $$Z_{in}=Z_{0}\sqrt{\frac{\mu_{r}}{\varepsilon_{r}}}\tanh\left[j\left(\frac{2\pi ƒd}{c}\right)\sqrt{\mu_{r}\varepsilon_{r}}\right]$$

(8) $$\mathrm{RL}(\mathrm{dB})=20\log\left|\frac{Z_{in}-Z_{0}}{Z_{in}+Z_{0}}\right|$$

where *Z_in_* and *Z*_0_ are the input characteristic and the free space impedance values, respectively; *ε_r_* and *μ_r_* are the complex permittivity and permeability, respectively; *c* is velocity, *f* is EM wave frequency in free space, and *d* is the absorber thickness. The results are shown in Figure 9. Due to the large particle size and higher impurity, the minimum RL of M0 is −8.1 dB at 5.7 GHz with a thickness of 5.0 mm, while the minimum RL of the M1 sample is −21.5 dB at 17.4 GHz with a thickness of 5.0 mm. The RL value of M2 reaches the highest value of −34.0 dB at 16.7 GHz with a thickness of 5.0 mm, and the effective frequency bandwidth is 2.3 GHz (4.8–5.4 GHz and 15.9–17.6 GHz). Similarly, the minimum RL value of M3 reaches −31.4 dB at 16.7 GHz with the same thickness, and the effective frequency bandwidth is 2.1 GHz (4.8–5.2 GHz and 15.9–17.6 GHz). This probably attributes both size effect and influence of silicates.

The thickness of the absorber significantly affects the microwave absorption performance. Therefore, the quarter-wavelength matching model (*λ*/4 model) is used to optimize the material thickness according to the best performance data. The relationship between peak frequency and material thickness (*t_m_*) can be described by Equation (9) [39]:

(9) $$t_{m}=\frac{n\lambda }{4}=\frac{nc}{4f_{m}\sqrt{\left|\mu_{r}||\varepsilon_{r}\right|}}$$

where *n* represents the uneven number, *λ* is the wavelength, and *ƒ_m_* is frequency. When an EM wave enters a material, two reflection microwaves are generated from the interfaces of absorber–metal and absorber–air. If the matching thickness of the absorber obeys Equation (9), the interference cancellation of EM waves occur at the interface of the air–absorber. The RL peaks are consistent with the *λ*/4 model when their thickness changes from 1.0 to 5.0 mm, as shown in Figure 9, indicating that the absorbing characteristics of all the samples conform to the *λ*/4 model.

**Figure 10:**
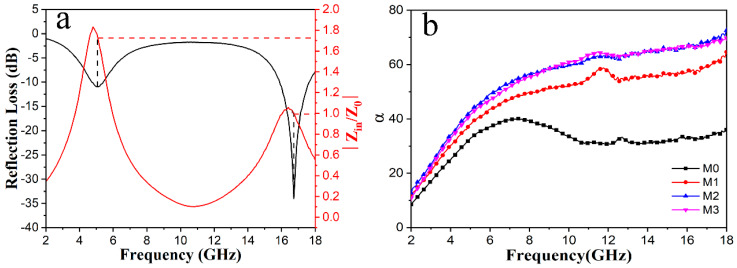
RL value and impedance matching curve of M2 with thickness of 5 mm (**a**), attenuation constant curves of M0, M1, M2, and M3 (**b**).

Strong EM wave dissipative capacity and impedance matching are the main factors for EM wave absorption materials. Impedance matching is required for EM waves to penetrate into materials instead of being reflected, which can be expressed as *Z* = |*Z*_in_/*Z*_0_|. When *Z* approaches 1, optimal impedance matching is obtained. Therefore, the smallest RL value can be obtained at 16.7 GHz corresponding *Z* close to 1 (see Figure 10a), indicating that M2 has a preferable impedance matching. Although the *Z* values were also close to 1 at 4.2 and 5.5 GHz, the lower *ε″* value results in weaker EM wave absorption capability.

The attenuation constant α reflects the EM wave loss ability of a material, and can be expressed by the following equation [40]:

(10) $$\alpha =\frac{\sqrt{2}\pi f}{c}\times \sqrt{(\mu {}^{\prime \prime }\varepsilon {}^{\prime \prime }-\mu {}^{\prime }\varepsilon {}^{\prime })+\sqrt{{\left(\mu {}^{\prime \prime }\varepsilon {}^{\prime \prime }-\mu {}^{\prime }\varepsilon {}^{\prime }\right)}^{2}+{\left(\mu {}^{\prime }\varepsilon {}^{\prime \prime }+\mu {}^{\prime \prime }\varepsilon {}^{\prime }\right)}^{2}}}$$

where *c* is microwave velocity in free space, and *f* is frequency. M0 demonstrates a lower *α* value because of its weak dielectric and magnetic loss capability. The *α* values of the M2 and M3 are remarkably higher than that of M1, implying their stronger attenuation capability for incident microwaves, as shown in Figure 10b.

Thus, ball-milling processing can improve RL values and expansion of the effective bandwidth. For example, the M3 RL value is close to M2, which could be attributed to its smaller particle size, higher defect density, and lower silicates content. The EM wave absorption performances of MPs and other related composites are shown in Table 5. Although the EM wave absorption properties of MPs are not excellent enough, the material preparation pathway (extracted from nickel slag) and the tunable absorbing properties by ball milling are still interesting. It should be affirmed that the improvements in EM wave absorption performance of MPs and recombination with other materials are worthy of further study.

**Figure 11:**
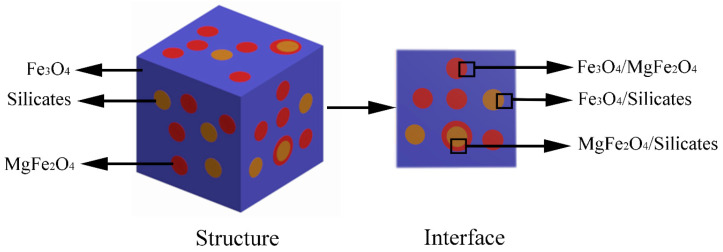
Schematic representation of the interface structure of MPs.

In short, the advantages of using as-prepared MPs to design MAM are as follows: (i) ball-milling can not only reduce particle size but also introduce numerous defects, which strengthens the dielectric relaxation; (ii) the multi-interfacial polarization induced between silicates and magnetite is beneficial for improving their microwave absorption performance; (iii) the synergy effect of dielectric loss and magnetic loss can be optimized to form good impedance matching. These descriptions are graphically shown in Figure 11.

## 4. Conclusions

This work demonstrates an inexpensive and environment-friendly method to prepare magnetite particles that can be used as MAM. Ball milling is used to tailor the MPs’ morphology and size—that which improves their microwave absorption properties—attributable to particle size effect and silicates’ dissociation. The results indicate that particle size and heterogeneous interface structures significantly affect MPs’ microwave absorbing properties. MPs milled for 6 h showed the lowest RL value −34 dB at 16.72 GHz with thickness of 5 mm; effective absorption bandwidth was about 2.3 GHz. As the ball-milling time increases to 12 h, the microwave absorption capacity decreases to −31.4 dB. This work provided insights into the preparation of MAM from slags and tunable absorbing properties by the ball-milling method.

## Figures and Tables

**Table 1:** Composition of nickel slag (wt%).

Component	Fe	Ca	Mg	Si	Ni	Co	Cu	S	Others
Content	36.22	3.75	8.94	14.28	0.65	0.12	0.27	0.63	11.49

**Table 2:** Average grain size (*δ*) and internal strain (*ε*) of MPs with different ball-milling time.

Samples	Average Grain Size (nm)	Internal Strain (%)
M0	155.7	0.098
M1	137.4	0.148
M2	101.1	0.162
M3	54.4	0.292

**Table 3:** Elemental composition of the oxidized slag (wt%).

	Fe	Si	Mg	Ca	O	Cu	Ni	Co	Al
1	5.53	26.02	7.56	5.65	53.3	0	0	0	1.94
2	62.82	0	3.11	0	30.82	0.73	1.54	0.44	0.54

**Table 4:** Resistivity values of M1, M2, and M3.

Samples	M1	M2	M3
Electrical resistivity (kΩ·cm)	9.63	6.245	7.71

**Table 5:** Microwave absorption properties of MPs and other related composites in the frequency range of 2–18 GHz.

Sample	Absorber and Content	Reflection Loss	Effective Frequency Bandwidth	Ref.
1	Fe_3_O_4_/Fe	−25.9 dB	4.2 GHz	[41]
2	Fe_3_O_4_/C	−20.6 dB	4.7 GHz	[35]
3	Fe_87.5_Si_7_A_l5.5_	−22.2 dB	6.6 GHz	[20]
4	Fe_3_O_4_@MnO_2_	−42.6 dB	6.6 GHz	[10]
5	Fe_3_O_4_/Cu	−53.4 dB	5.84 GHz	[42]
6	(Fe_3_O_4_/ZnO)@C	−40.0 dB	6.5 GHz	[8]
7	Fe_3_O_4_@SiO_2_@PPy	−40.9 dB	6.88 GHz	[7]
8	Fe_3_O_4_@SiO_2_@ZnO	−24.4 dB	8.86 GHz	[43]
9	Fe_3_O_4_/Silicates	−34.0 dB	2.3 GHz	This work
